# Altered cellular redox status, sirtuin abundance and clock gene expression in a mouse model of developmentally primed NASH^☆^

**DOI:** 10.1016/j.bbalip.2016.03.026

**Published:** 2016-07

**Authors:** Kimberley D. Bruce, Dawid Szczepankiewicz, Kiran K. Sihota, Manoj Ravindraanandan, Hugh Thomas, Karen A. Lillycrop, Graham C. Burdge, Mark A. Hanson, Christopher D. Byrne, Felino R. Cagampang

**Affiliations:** a Institute of Developmental Sciences, Faculty of Medicine, University of Southampton, Southampton General Hospital, Southampton, UK; b University of Colorado Anschutz Medical Campus, Endocrinology, Metabolism and Diabetes, Aurora, USA; c Poznań University of Life Sciences, Department of Animal Physiology and Biochemistry, Poznań, Poland; d Centre for Biological Sciences, Institute of Developmental Sciences, Faculty of Natural and Environmental Sciences, University of Southampton, Southampton, UK

**Keywords:** NAFLD, non-alcoholic fatty liver disease, NASH, non-alcoholic steatohepatitis, HF, high fat, C, control, sirt1, sirtuin 1, sirt3, sirtuin 3, Clock, circadian locomotor output cycles kaput, Per2, Bmal1 (also called Arntl1), period 2, Cry2, cryptochrome 2, RORα, retinoic acid receptor-related orphan receptor alpha, Srebp1c, sterol regulatory element binding protein-1c, NAD, nicotinamide adenine dinucleotide, OPN, osteopontin, High fat, Maternal diet, Fatty liver, Development, Aging, Circadian

## Abstract

**Background:**

We have previously shown that high fat (HF) feeding during pregnancy primes the development of non-alcoholic steatohepatits (NASH) in the adult offspring. However, the underlying mechanisms are unclear.

**Aims:**

Since the endogenous molecular clock can regulate hepatic lipid metabolism, we investigated whether exposure to a HF diet during development could alter hepatic clock gene expression and contribute to NASH onset in later life.

**Methods:**

Female mice were fed either a control (C, 7% kcal fat) or HF (45% kcal fat) diet. Offspring were fed either a C or HF diet resulting in four offspring groups: C/C, C/HF, HF/C and HF/HF. NAFLD progression, cellular redox status, sirtuin expression (*Sirt1*, *Sirt3*), and the expression of core clock genes (*Clock*, *Bmal1*, *Per2*, *Cry2*) and clock-controlled genes involved in lipid metabolism (*Rev-Erbα*, *Rev-Erbβ*, *RORα*, and *Srebp1c*) were measured in offspring livers.

**Results:**

Offspring fed a HF diet developed NAFLD. However HF fed offspring of mothers fed a HF diet developed NASH, coupled with significantly reduced NAD^+^/NADH (p < 0.05, HF/HF vs C/C), Sirt1 (p < 0.001, HF/HF vs C/C), Sirt3 (p < 0.01, HF/HF vs C/C), perturbed clock gene expression, and elevated expression of genes involved lipid metabolism, such as Srebp1c (p < 0.05, C/HF and HF/HF vs C/C).

**Conclusion:**

Our results suggest that exposure to excess dietary fat during early and post-natal life increases the susceptibility to develop NASH in adulthood, involving altered cellular redox status, reduced sirtuin abundance, and desynchronized clock gene expression.

## Highlights

• Offspring of mothers fed a high fat diet show severe fatty liver in later life.
• HF feeding is associated with altered cellular redox status and reduced sirtuin gene expression.
• HF feeding desynchronises the expression of core clock genes and lipogenic transcription factors.
• Exposure to a HF diet during development causes changes in liver metabolism that precede severe fatty liver disease.

## 1 Introduction

Non-alcoholic fatty liver disease (NAFLD) is currently the most common cause of chronic liver disease worldwide, and is present in a third of the general population and the majority of individuals with obesity and type 2 diabetes [1], [2]. In its milder form, NAFLD is characterized by excessive intra-hepatocyte triglyceride (TG) accumulation (hepatic steatosis). In advanced stages steatosis is coupled with inflammation and termed non-alcoholic steatohepatits (NASH) [3], which can eventually result in liver failure. NAFLD is commonly associated with older individuals; however, recent evidence suggests that NAFLD is increasingly common in young adults, children and adolescents [4], [5], [6]. The precise mechanisms underlying the premature onset of liver disease are unclear.

A substantial body of evidence suggests that early life is a critical period of plasticity, in which the developing organism physiologically adapts to its surrounding environment. In some cases this may be advantageous [7], [8], however, imbalanced nutrition during early life may also have deleterious effects on the development of key metabolic organs. This is particularly true of the liver, which undergoes important maturation stages during late gestation and early postnatal life. Hence, the liver is highly susceptible to chronic maternal high fat (HF) diets, which can lead to a NAFLD phenotype in offspring independent of maternal and offspring obesity [9]. In addition, we have previously shown in mice, that exposure to a high fat (HF) diet during gestation and lactation primes the development of the severe form of fatty liver (NASH) in adult offspring, involving impaired mitochondrial function and increased expression of genes involved in lipogenesis [10]. Further studies support the notion that early HF exposure can prime the onset of NAFLD in adulthood. In a rat model of high fat feeding during pregnancy only, obese mothers gave rise to offspring with altered transcriptional and epigenetic regulation of key regulators of fatty acid oxidation such as peroxisome proliferator-activated receptor (PPAR)α [11]. Similarly offspring of HF fed dams have shown increased expression of the lipogenic transcription factor, *Srebp1c* [12], [13].

Nutritional status is constantly being assessed by transcriptional networks that control lipid homeostasis [14]. In mammals, one such network is the endogenous molecular clock network, which mediates transcriptional changes controlling a number of metabolic outputs [15]. While the suprachiasmatic nucleus (SCN) of the hypothalamus is the central pacemaker, molecular clocks also exist in peripheral tissues such as the liver where they can be entrained by local nutrient availability [16]. The core components of the clock network *Clock*, *Bmal1*, *Per1*, *Per2*, *Cry1 and Cry2*, self-regulate through transcriptional feedback loops resulting in 24-hour oscillatory patterns of gene expression [17], [18]. Interestingly, genetic disruption of the clock genes and changes in the canonical 24-hour oscillatory patterns can lead to fatty liver disease [19], [20]. This is likely due to the fact that the core clock genes transcriptionally regulate downstream clock controlled genes (CCGs) with important roles in hepatic lipid metabolism. For example, *Bmal1* has been shown to directly regulate CCGs involved in hepatic carbohydrate and lipid metabolism [21]. In addition, the *Rev-Erb and ROR* nuclear transcription factors are able to activate and repress *Bmal1* expression respectively, thus fine tuning the circadian regulation of metabolic genes [22]. The *Rev-Erb* isoforms, previously considered as accessory components of the clock system, have been shown to coordinate a number of genes involved in lipid metabolism [23]. Recent studies using *Rev-Erb* agonists have revealed alterations in the nycthemeral (day vs night) patterns in both behaviour and hepatic gene expression [24]. Importantly, the maternal environment has the capacity to alter clock gene expression in the developing liver, causing changes that persist postpartum [25]. For example, in non-human primate liver, exposure to a maternal high-fat diet significantly alters expression of fetal hepatic *Npas2*, a paralog of the clock transcription factor [26]. In rodents, offspring of obese dams show reduced hepatic expression of both circadian and metabolic genes, which is associated with altered mRNA dynamics [11]. More recently it has been shown that maternal obesity interacts with an obesogenic post-weaning diet to disrupt the canonical rhythmicity of gene expression in the liver, and alters the DNA methylation levels at both the *Bmal1* and *Per2* promoters [27].

Recent findings suggest that the *Clock* and *Bmal1* heterodimer sense the metabolic status of the cell via a functional interaction with the NAD^+^ dependent deacetylase SIRT1 [28], [29]. SIRT1 belongs to a class of sirtuin proteins with roles in circadian rhythms, mitochondrial metabolism, and aging [30], [31], [32]. Moreover, recent findings have shown that SIRT3, a mitochondrial protein deacetylase, is down-regulated in response to maternal fat exposure and is involved in the development of fatty liver disease [33], [34]. Thus, it is possible that SIRT1 and SIRT3 functionally link mitochondrial metabolism, lipid homeostasis, circadian biology and age-associated metabolic decline.

In light of these findings we tested the hypothesis that HF feeding during development could contribute to the priming of severe fatty liver disease, through altered circadian biology, clock gene expression, and altered regulation of CCGs with key roles in lipid homeostasis.

## 2 Methods

### 2.1 Animal model

All animal procedures were carried out in accordance with the United Kingdom Animals (Scientific Procedures) Act 1986 and were approved by the local ethics review committee. Female C57/BL6J mice were maintained under a 12-hour light/dark cycle (lights on at 07.00 h), and at a constant temperature of 22 ± 2 °C with food and water available ad libitum. Dams were randomly allocated to one of two diets: control (C; 7% kcal fat; Special Dietary Services, UK), or a high fat diet (HF; 45% kcal fat; Special Dietary Services, UK). We have previously used this HF diet to bring about an obese phenotype in both the pregnant dams and their offspring [10]. Dams were fed the designated diet 6 weeks pre-pregnancy, through to pregnancy and lactation. Pregnant dams were allowed to deliver their pups, and litter size was standardized to six pups (three males and three females, whenever possible) to ensure that no litter was nutritionally biased. Offspring were randomly allocated to either the C or HF diet at weaning at 3 weeks of age resulting in 4 offspring groups; C/C, C/HF, HF/C and HF/HF. These offspring were fed the diets for the next 12 weeks. At 15 weeks of age, male offspring were killed by cervical dislocation at 2 time points during the 24-hour light-dark period, at 3 pm (Zeitgeber time 8 or ZT8; 8 h into the light or day period) and at 3 am (ZT20; 8 h into the dark or night period). Liver tissue was immediately frozen in liquid nitrogen and stored at − 80 °C or paraffin embedded for histological analysis.

### 2.2 Day-night changes in food intake and energy expenditure

Food intake and energy expenditure were recorded over a 24-hour light–dark cycle in the four offspring groups (C/C, C/HF, HF/C and HF/HF, n = 6–10 per offspring group) using a closed modular indirect calorimetric system at 12 weeks of age (Oxylet, Panlab SLU, Spain). Oxygen consumption (*V*O_2_) and carbon dioxide production (*V*CO_2_) were recorded at 5-min intervals using a computer-assisted data acquisition program (Metabolism; Panlab SLU, Spain) over a 24-hour period. From this, the animal’s energy expenditure (EE; in kcal/day/body weight^0.75^) was calculated. Food intake was continuously measured using an extensiometric food weight transducer device (Panlab SLU, Spain). To determine day and night differences in food intake and energy expenditure between the offspring groups, the mean values were calculated during the light and dark phases of the cycle for each mouse in each treatment groups.

### 2.3 Glucose tolerance test

Glucose tolerance test was performed after an overnight fast at 14 weeks of age. Fasting glucose concentration was measured in whole blood obtained from the tail vein before the fasted mice were intraperitoneally injected with d-glucose (2 g/kg mouse body weight), and blood glucose concentration was measured using a glucometer (Aviva Accu-Chek, Roche Diagnostics Ltd., UK) at 15, 30, 60 and 120 min.

### 2.4 Evaluation of steatosis by point counting

Evaluation of steatosis was done by point counting. Previous studies have shown that the point counting method of evaluating steatosis had a strong and significant correlation to hepatic triglyceride levels [53]. Briefly, captured digital images of liver sections stained with hematoxylin and eosin (H&E) were projected on an LCD monitor. For each H&E stained liver sections, sequential images were taken at 20 × magnification across a section (avoiding large vessels). A total of 3 stained sections were scanned per animal (n = 4 animals per treatment group). Using a computer software FIJI (http://fiji.sc/Fiji), a 10 × 10 grid system of 100 test points (PT) was superimposed on the image field. The percentage volume density of hepatic steatosis (Vv[steatosis, liver]%) was then estimated as the ratio of the points hitting the vesicles of fat (Pp) compared to the number of test points: Vv[steatosis, liver]% = (Pp[steatosis] / PT) × 100.

### 2.5 Histological analysis

The Kleiner scoring system was used to assess the severity of NAFLD [35]. An activity score (AF) was generated by adding the individual score for necroinflammatory features; steatosis (< 5% = 0, 5–33% = 1, 33–66% = 2, > 66% = 3); ballooning (none = 0, few = 1, prominent = 2); and lobular inflammation (none = 0, < 2 foci = 1, 2–4 foci = 2, > 4 foci = 3). A score of < 3 correlates with mild NAFL, a score of 3–4 correlates with moderate NAFL and a score of 5 or more correlates with NASH. The average score for each histological characteristic in each group was used as previously [10].

### 2.6 NAD/NADH assay

NAD^+^ and NADH were extracted from left lobe liver samples and quantified using an enzymatic calorimetric method (Enzychrom, BioAssay Systems, CA, USA) according to the manufacturer's instructions. Both absolute levels and NAD^+^/NADH ratios are presented.

### 2.7 Plasma lipid assay

Day (ZT8) and night (ZT20) triglyceride levels in plasma samples (n = 5 per group, per time point) was measured with a commercial enzymatic colorimetric method (cholesterol 50 kits; Sigma, Poole, UK), and plasma NEFA was measured by an enzymatic method (Alpha Laboratories, Eastleigh, UK).

### 2.8 Relative gene expression

Total RNA was isolated from 0.025 g of left lobe of the liver from 15 week-old offspring (n = 5 per time point and dietary group) using Trifast reagent (Peqlab, Germany) according to the manufacturer's instructions. RNA quantification, quality, and integrity were determined via (260/230 and 260/280 ratios and concentrations) a NanoDrop spectrophotometer (Thermo Fisher Scientific, UK) and agarose gel electrophoresis. Total RNA (1 μg) was reverse transcribed to cDNA using reverse M-MLV transcriptase (Promega, UK). Samples were then incubated at 37 °C for 1 h, 42 °C for 10 min, followed by an enzyme activation step at 75 °C for 10 min. The cDNA synthesis was then diluted to a concentration of 5 ng/μL before amplification. Expression of the *Sirt1*, *Sirt3*, the core clock genes (*Clock*, *Bmal1*, *Cry1*, *Cry2*, *Per2*), and the lipogenic transcription factors *Rev-Erbα*, *Rev-Erbβ*, *RORα*, *and Srebp1c* were determined at ZT8 and ZT20, otherwise referred to as day and night, by quantitative real time reverse transcriptase polymerase chain reaction (qRT-PCR; Applied Biosystems 7500 Fast Real-time PCR Thermal Cycler). Expression of *OPN* was determined at ZT8 only. We used the housekeeping genes (HKG) *YWHAZ* and *βactin* to determine relative gene expression, since they were the most stably expressed gene following challenge with HF diets and at different circadian time points [36]. PCR amplification was performed for 50 cycles. Following an initial enzyme activation step for 10 min at 95 °C, each cycle consisted of denaturation for 15 s at 95 °C, annealing for 30 s at 50 °C and 15 s at 72 °C. Analysis of duplicated samples determined that the intra-assay coefficients of variability (CV) was > 5%.

### 2.9 Statistical analysis

Data are expressed as mean ± SEM, and a significant difference was accepted at p < 0.05. For 24-hour metabolic phenotype measurements, day (light period) or night (dark period) area under the curve (AUC) analysis was performed on individual animals. A one way analysis of variance (ANOVA) was performed between offspring groups with Bonferroni post-hoc analysis for multiple comparisons, to determine the groups which significantly differed. For blood analysis, mean day (ZT8) and night (ZT20) values were analyzed by ANOVA and Bonferroni post-hoc tests. For gene expression analysis, mean delta delta CT values for each offspring group at either day (ZT8) or night (ZT20) were tested for significant difference using ANOVA followed by Bonferroni post-hoc analysis.

## 3 Results

### 3.1 Food intake and energy expenditure is perturbed in 15 week old male offspring exposed to high fat diets during development

**Fig. 1:**
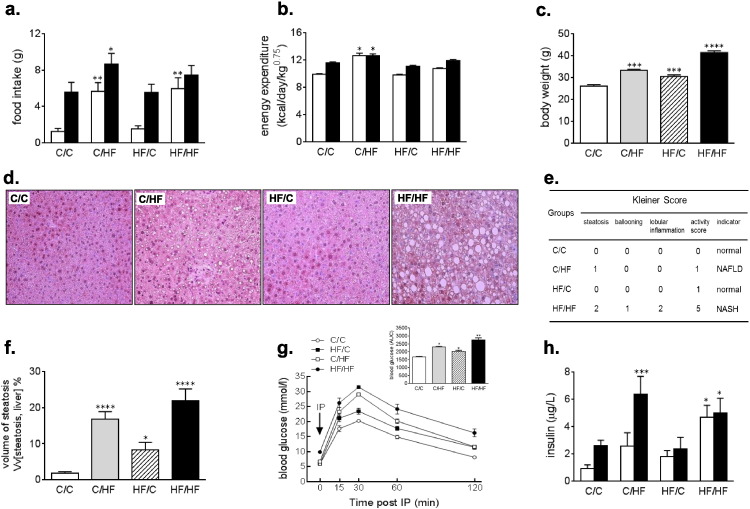
15 week old male offspring exposed to high fat diets during development and adulthood have perturbed nycthemeral (day vs night) rhythms of food intake and energy expenditure, and also develop severe fatty liver and imbalanced hepatic cellular redox status. (a.) Total amount of food intake of male offspring from each dietary group, during the 12 h light (daytime, open bars) and 12 h dark (nighttime, closed bars) periods. (b.) Energy expenditure levels in male offspring from each dietary group, during the 12 h light (open bars) and 12 h dark periods (closed bars). (c.) Body weight (recorded at ZT8) of the offspring at 15 weeks of age. (d.) Percentage of steatosis in liver sections determined by point counting (e.) Hematoxylin and eosin stained liver sections from 15 week old male offspring. C/C and HF/C offspring show normal liver architecture. C/HF offspring livers exhibit lipid accumulation. HF/HF livers contain significant lipid accumulation and ballooning degeneration. (f.) Kleiner scores for offspring liver (n = 4 for each offspring group), show that C/C and HF/C are histologically normal, C/HF show a histological phenotype similar to human non-alcoholic fatty liver (NAFL), and HF/HF offspring liver have the appearance of non-alcoholic steatohepatitis (NASH). (g.) Fasting blood glucose over 2 h following bolus intraperitoneal (IP) injection of glucose (inverted arrow). Inset histogram depicts the area under the curve (AUC) levels over the 2-hour monitoring period. (h.) Circulating insulin levels during the 12 h light (daytime, open bars) and 12-hour dark (nighttime, closed bars) periods. For each analysis offspring groups were compared against C/C, where *p < 0.05, **p < 0.01, ***p < 0.001 or ****p < 0.0001 vs C/C.

To determine the effect of increased fat exposure on circadian physiology, we measured nycthemeral patterns of food intake and energy expenditure (EE) in adult offspring. Control offspring showed a nocturnal phenotype and consumed food mainly during the subjective night (Fig. 1a). The canonical night-time feeding behavior was also observed in the HF/C offspring. In contrast, both offspring groups fed a HF diet during adulthood ate significantly more during the day than the control offspring (Fig. 1a; p < 0.01, C/HF or HF/HF vs C/C). Although total food intake was greatest in the C/HF offspring, these offspring also had the significantly higher EE in both the day and the night than other offspring groups (Fig. 1b; p < 0.05, C/HF vs C/C). It is plausible to suggest that such an increase in EE offsets the increased caloric intake observed in the C/HF offspring, and limits further weight gain. This may partially explain why the HF/HF offspring, which lack increased EE, are significantly heavier than other offspring groups (Fig. 1c; p < 0.001, C/HF vs C/C; p < 0.0001, HF/HF vs C/C). Although speculative, the maternal HF challenge may alter metabolic capacity in the offspring. In the HF/HF offspring this prevents an adaptive increase in EE that would otherwise limit further weight increase, while in the HF/C this manifests as significantly higher body weight in the absence of increased food intake.

### 3.2 Offspring exposed to HF diets during development are primed to develop severe fatty liver in adulthood

We have previously shown that female offspring exposed to high fat diets during development are primed to develop severe fatty liver disease, characterized by micro and macrovesicular steatosis, ballooning degeneration, and inflammation [10]. In the present study, we asked whether male offspring developed a similar condition. Steatosis was directly quantified by point counting to show that while HF/C offspring had increased fat accumulation compared to control, fat accumulation was further increased in the C/HF offspring, and further still in their HF/HF counterparts (Fig. 1d). Histological analysis and Kleiner scoring methods were used to compare the severity of liver disease between offspring groups. Histological analysis confirmed that while offspring exposed to a HF diet during adulthood (C/HF) displayed marked hepatic lipid accumulation, offspring exposed to HF diets during development and later life (HF/HF) displayed a severe liver histopathology akin to human NASH (Fig. 1e). In confirmation, histological scoring of liver sections revealed that livers from the HF/HF offspring had scores of 5 or greater, which were consistent with the features of NASH (Fig. 1f).

Glucose tolerance tests (GTT) were performed to determine the effect of HF feeding on glucose homeostasis. GTT revealed that baseline fasting glucose levels were significantly higher in the HF/HF offspring compared to the other offspring groups vs C/C (Fig. 1g p < 0.01). Following (intraperitoneal) IP injection of glucose, circulating glucose levels remained significantly elevated, even at after 2 h post-IP in both groups of offspring exposed to a HF diet during development (p < 0.05 in HF/C and p < 0.01 in HF/HF vs C/C) and those fed a HF diet in later life (p < 0.05, C/HF vs C/C). Since this elevated blood glucose and reduced glucose clearance is associated with insulin resistance we measured day and night circulating insulin levels. (Fig. 1h). Insulin was significantly higher in the C/HF offspring during the night (p < 0.001, C/HF vs C/C). However, HF/HF offspring were hyperinsulinemic during both the day and night, and the nycthemeral pattern of insulin production was seemingly abolished (p < 0.001, HF/HF vs C/C).

**Fig. 2:**
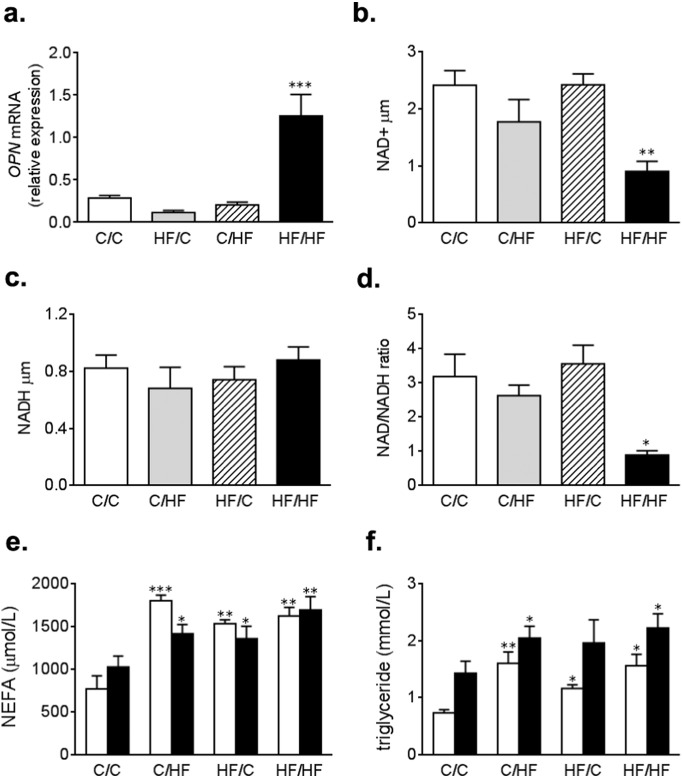
As a measure of fibrosis (a.) OPN gene expression was measured in offspring liver. Concentration of pyridine nucleotides (b.) NAD^+^, (c.) NADH in offspring liver (left lobe), and (d.) the NAD^+^/NADH ratio in offspring liver. Circulating levels of (e.) non-esterified fatty acids (NEFAs) and (f.) triglycerides were determined from mouse plasma taken during the light (daytime at ZT8, open bars) or dark (nighttime at ZT20, closed bars) periods (for each analysis offspring groups were compared against C/C, where *p < 0.05, **p < 0.01 or ***p < 0.001 vs C/C.

Since murine models readily develop NAFLD, but not NASH, we set out to measure *OPN* expression, a marker of fibrosis and severe fatty liver disease in offspring liver (Fig. 2a). We found that OPN expression significantly increased the livers of HF/HF offspring (p < 0.001 in HF/HF vs C/HF), but not in any other offspring groups. This suggests that the combination of high fat feeding during both early and post-natal life, predisposes to the development of a more severe fatty liver disease akin to human NASH.

### 3.3 Offspring exposed to a HF diet during development and adulthood have imbalanced cellular redox status and altered metabolic state

To determine whether HF feeding during development resulted in alterations in cellular redox status, we measured the availability of NAD^+^ and NADH in offspring liver. Offspring exposed to a HF diet during both development and adulthood (HF/HF) had significantly reduced hepatic NAD^+^ (Fig. 2b; p < 0.01, HF/HF vs C/C). In contrast, hepatic NADH concentrations were similar in all offspring groups (Fig. 2c). Consequently, the NAD^+^/NADH ratio was significantly lower the in HF/HF offspring compared to all other offspring groups (Fig. 2d; p < 0.05, HF/HF vs C/C).

### 3.4 Offspring exposed to HF diets have altered lipid homeostasis

To determine the effect of HF feeding on circadian lipid homeostasis, we measured circulating non-esterified fatty acids (NEFA) and triglycerides (TG) in each offspring group at a specific time during the day (ZT8), or night (ZT20). Offspring exposed to a HF diet at any life stage, had increased daytime circulating NEFA compared to C/C offspring (Fig. 2e; p < 0.001, C/HF vs C/C; p < 0.01, HF/C or HF/HF vs C/C). Similarly, daytime TG levels were significantly higher in all offspring fed a HF diet (Fig. 2f; p < 0.01, C/HF vs C/C; p < 0.05, HF/C or HF/HF vs C/C). Moreover, offspring fed a HF diet in the post-natal period showed an increase in nighttime TGs compared to control offspring (p < 0.05, C/HF or HF/HF vs C/C).

### 3.5 Hepatic sirtuin and core clock protein expression is perturbed in offspring exposed to a HF diet during early life and adulthood

**Fig. 3:**
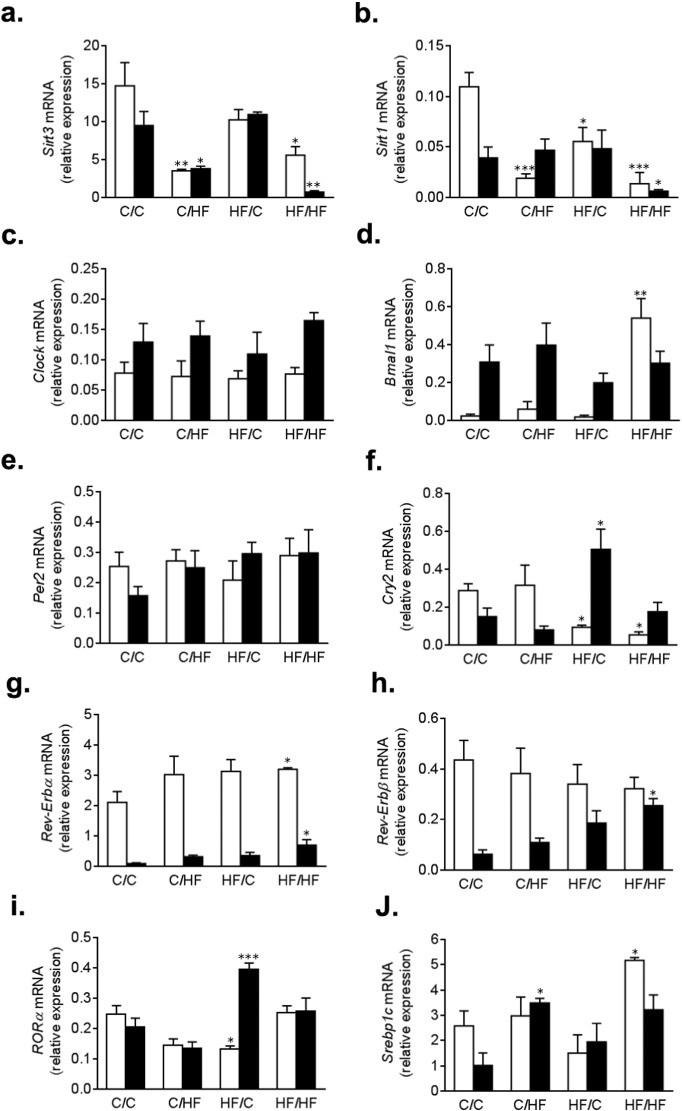
Mean relative hepatic mRNA expression of the sirtuins (a.) *Sirt1* and (b.) *Sirt3*; of genes involved in co-ordinating circadian rhythms (c.) *Clock*, (d.) *Bmal1*, (e.) *Per2*, and (f.) *Cry2*; and downstream lipogenic transcription factors (g.) *Rev-Erbα*, (h.) *Rev-Erbβ*, (i.) *RORα*, and (j.) *Srebp1c* during the light (daytime at ZT8, open bars) or dark (nighttime at ZT20, closed bars) periods. For each analysis offspring groups were compared against C/C, where *p < 0.05, **p < 0.01 or ***p < 0.001 vs C/C.

We had previously shown that mitochondrial function is impaired in offspring exposed to a HF diet during development [10]. Hence we determined the gene expression of mitochondrial *Sirt3* following HF feeding. *Sirt3* was significantly reduced in livers of offspring that had been fed a HF diet during adulthood (Fig. 3a; p < 0.01, C/HF vs C/C), corresponding to a − 3.8 fold decrease (Table 1). Nighttime expression of *Sirt3* was further reduced (− 12.5 fold, Table 1) in offspring that had been exposed to a HF diet during both development and adulthood (p < 0.01, HF/HF vs C/C). However, expression of *Sirt3* was not reduced in the livers of offspring that had been exposed to a HF diet during development only (i.e. the HF/C group). In contrast, *Sirt1* expression was reduced by − 1.9 (Table 1) during the day in the livers of offspring exposed to a HF diet during the developmental period (Fig. 3b; p < 0.05, HF/C vs C/C.), a decline that was exacerbated by post-natal HF feeding, showing 5.7 and 8 fold reduction (Table 1) in the C/HF and HF/HF offspring, respectively (Fig. 3b; p < 0.001, C/HF or HF/HF vs C/C.). In addition, to validate the reduction in *Sirt1* and *Sirt3* gene expression, we measured protein levels of SIRT1 and SIRT1 (Supplemental Fig. 1a and b, respectively) to show that HF diets reduce significantly reduce sirtuin abundance, with the greatest effect being observed in the HF/HF offspring (p < 0.001 HF/HF vs C/C).

Since SIRT1 interacts with CLOCK in an NAD^+^ dependent manner [18], [28], we measured the expression of core clock genes and CCGs with key roles in lipid homeostasis. Here, we show that *Clock* transcript levels were robustly expressed in livers of all offspring (Fig. 3c). In contrast, hepatic daytime gene expression of *Bmal1* (Fig. 3d) showed a 22 fold increase (Table 1) in offspring that have been fed a HF diet during development and adulthood, compared to control offspring (p < 0.01; HF/HF vs C/C). In addition, *Bmal1* expression, which showed a 13.75, 6 and 8.35 fold increase in the day in the C/C, C/HF and HF/HF offspring respectively, was reversed in the HF/HF offspring (Table 2; − 2.2 fold decrease in HF/HF).

CRY2 forms a functional heterodimer with PER2 which has an inhibitory effect on CLOCK/BMAL1. Interestingly, while the hepatic expression of *Per2* remains unperturbed following HF feeding (Fig. 3e), daytime expression of *Cry2* was significantly altered in offspring exposed to a HF diet (Fig. 3f; p < 0.05, HF/C or HF/HF vs C/C). The nycthemeral pattern was also altered in the HF/C and HF/HF offspring, and showed higher expression in the night (Table 2; 5.18 and 2.4 fold respectively), compared to higher expression in the day on both the C/C and HF/HF offspring.

We next determined the expression of CCGs with important roles in lipid metabolism. Specifically, the nighttime expression of both *Rev-Erbα* and *Rev-Erbβ* was significantly increased in the HF/HF offspring compared to control (Fig. 3g and h, respectively; p < 0.05, HF/HF vs C/C). In addition, the daytime expression of *Rev-Erbα* was also significantly increased compared to control (Fig. 3g; p < 0.05, HF/HF vs C/C). The expression *Rev-Erbβ* is normally much higher in the day than in the night (Table 2; − 6.73 in C/C). However, with increasing exposure to a HF diet, the nycthemeral pattern of *Rev-Erbβ* expression is lost (Table 2; − 3.22, − 1.59, − 1.23 fold in C/HF, HF/C and HF/HF, respectively). The orphan nuclear receptor *RORα*, also a key modulator of lipid metabolism, was similarly perturbed in the HF/C offspring in the day (Fig. 3i; p < 0.05, HF/C vs C/C) and night (Fig. 3i; p < 0.001, HF/C vs C/C). Both *Rev-Erb and RORα* may regulate lipid metabolism through the lipogenic transcription factor *Srebp1c.* Expression of this gene was increased in livers from both the C/HF and HF/HF offspring (Fig. 3j; p < 0.05, C/HF and HF/HF vs C/C), consistent with increased hepatic fat accumulation in these groups.

## 4 Discussion

**Fig. 4:**
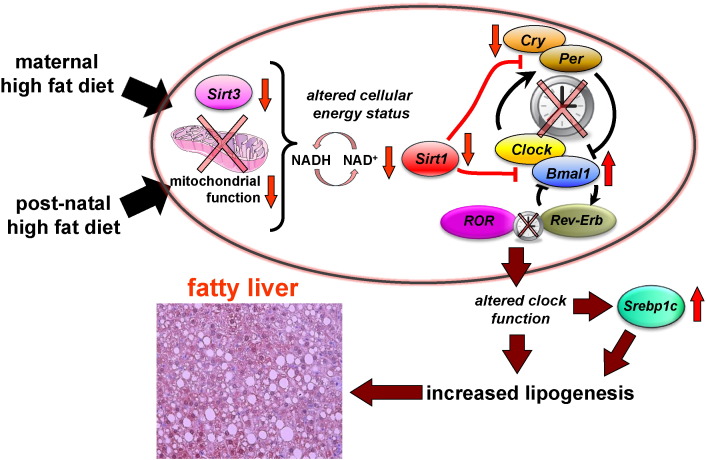
Hypothetical schematic representation of how maternal high fat and post-natal high fat (HF) diet alters sirtuin and clock gene expression leading to severe hepatic steatosis in offspring liver. A HF diet during both early and post-natal life reduces sirtuin levels and alters cellular energy status (reduction in NAD^+^ levels and NAD/NADH ratio). This is associated with desynchronization of circadian clock gene activity and upregulation of downstream lipogenic transcription factors such as *Srebp1c* to developmentally prime the lipogenic pathway, resulting in further up-regulation of fat accumulation.

Our findings suggest that the developmental priming of severe fatty liver involves a complex molecular pathology, involving alterations in circadian biology and lipogenesis. Specifically, we show that increased fat intake during development is associated with disruptions in circadian physiology, cellular redox status, sirtuin abundance, and transcription of core clock genes and down-stream lipogenic transcription factors (a schematic representation of this is illustrated in Fig. 4). These molecular changes appear to persist until adulthood, and may have deleterious effects on hepatic lipid homeostasis, increasing the likelihood of developing severe fatty liver disease in adulthood.

Since a growing number of young individuals are exposed to diets that are rich in fat during critical periods of development, it is likely that excess fat intake in early life contributes to the “primed” onset of NAFLD in young adults [37]. In this study we aimed to further understand the mechanisms underlying developmentally primed severe fatty liver disease. Here, we show that feeding a HF diet leads to NAFLD, however, the livers of male offspring of HF-fed mothers who are also given a HF diet in post-natal life (HF/HF offspring) show features pertaining to a more severe NASH-like phenotype. These results recapitulate our previous findings in female offspring [10], and moreover, demonstrate that the developmental priming of severe fatty liver is not sex-specific.

To further understand the pathogenesis of developmentally primed metabolic disorder, it is useful to examine differences in circadian physiology between offspring groups. Both groups of offspring fed a HF diet in the post-natal period (HF/HF and C/HF) show changes in metabolism akin to pre-clinical metabolic disorder, such as hyperglycemia, hyperlipidemia, increased food intake, and fatty liver disease. However, there are a number of interesting phenotypic differences between these two offspring groups. Firstly, the C/HF mice display increased insulin levels at night, when food intake and insulin levels are expected to concomitantly rise, albeit elevated due to the macronutrient composition of the HF diet. In contrast, the HF/HF mice show hyperinsulinemia in both the day and the night, highlighting marked changes in circadian physiology following developmental HF exposure, and suggesting that these offspring are insulin resistant, a known factor in the pathogenesis of fatty liver disease. In addition, the C/HF offspring also show increased energy expenditure and remain glucose tolerant. It is plausible that this is an adaptive response to increased energy intake and fat consumption in a heavier, yet healthy organism. These adaptations, namely increased EE may offer protection against increased ectopic lipid accumulation and severe fatty liver disease. In contrast the HF/HF offspring fail to increase energy expenditure in response to increased energy intake, and also exhibited pronounced hyperglycemia, at both baseline and post-glucose bolus, which may be indicative of a developmentally programmed inability to respond to nutritional excess. Thus the HF/HF are less able to response to increased fat consumption, and unlike their C/HF counterparts, who were fed the same diet but did not experience a developmental HF challenge, are not protected against increased ectopic lipid accumulation and continue to develop severe fatty liver disease.

Reduced metabolic capacity in the HF/HF offspring is consistent with our previous work, which showed that developmental fat exposure can impair mitochondrial electron transport chain (ETC) function and metabolic capacity in offspring livers [10]. In further support, in the present study we observe impaired redox status and significantly reduced *Sirt1* and *Sirt3* gene expression in the HF/HF offspring. Importantly, SIRT3 is thought to interact with subunits of complexes I and II of the electron transport chain (ETC) directly, leading to increased mitochondrial efficiency [38]. Thus, it is plausible to suggest that prolonged exposure to diets containing excessive amounts of fat could alter nutrient sensing via mitochondrial sirtuins and directly influence mitochondrial capacity and disease susceptibility.

Our findings are consistent with recent studies implicating SIRT3 as a key player in fatty liver disease pathogenesis [39]. Interestingly, SIRT3 mediated lipotoxicity is thought to involve hyperacetylation of mitochondrial proteins and dysregulation of the electron transport chain (ETC) enzymes, leading to protein oxidation and impaired mitochondrial function [40]. Since ETC enzyme function is impaired in offspring exposed to a HF diet during development, we suggest that SIRT3 reduction plays an important role in developmentally primed fatty liver. How SIRT3 becomes reduced is unclear, however, SIRT3 reduction is often coupled to reduced NAD^+^ levels [39], and supplementation with the NAD^+^ precursor nicotinamide ribose activates SIRT3 and enhances oxidative metabolism to protect against HF-induced metabolic abnormalities [41]. Here, we show that NAD^+^ is markedly reduced in the livers of offspring from obese mothers that were also fed a HF diet in later life, suggesting that depleted NAD^+^ levels in these HF/HF offspring contributes to reduced SIRT3 abundance. Whether increased fat intake activates NAD^+^ consuming enzymes to deplete the cellular pool of NAD^+^, altering redox balance and reducing sirtuin abundance remains to be tested. Nonetheless, in support of this premise *Sirt1* gene expression, for which NAD^+^ is also a rate-limiting co-substrate, is also down-regulated in the livers of HF/HF offspring.

Multiple cellular processes play in important role in maintaining the NAD^+^/NADH ratio. For example, during glycolysis, beta-oxidation, and the citric acid cycle NAD^+^ is reduced to NADH. Once in the mitochondria NADH is oxidized by the electron transport chain enzymes during oxidative phosphorylation. Since we have previously shown that the HF/HF offspring exhibit impaired oxidative metabolism through reduced electron transport chain activity (Bruce et al. [10]), it is possible to suggest that the HF/HF offspring are in a state of redox imbalance, and although their livers are able to readily reduce NADH during catabolic redox reactions (in response to increased fat intake), their ability to replenish NAD^+^ reserves is lacking due to impaired oxidative capacity. Conversely, the C/HF offspring, which do not have an impaired oxidative metabolism, are able to maintain redox balance even during increased food intake. Similarly, the HF/C offspring, which may also have developmentally impaired electron transport chain enzymes, are not burdened with the post-natal challenge of a high fat diet, and thus are also able to maintain a favorable NAD^+^/NADH ratio.

As the liver ages it is more prone to metabolic changes such as ectopic lipid accumulation [42]. This is consistent with growing evidence suggesting that fatty liver disease is more prevalent in older individuals [43], [44]. Here, we show that HF feeding during early and later life is associated with depleted NAD^+^ reserves and reduced sirtuin abundance, both established hallmarks of metabolic aging [31]. Importantly, offspring exhibiting these hallmarks also display serve fatty liver disease at a fairly young age (15 weeks old). Therefore, we suggest that HF exposure during development may impose chronic metabolic stress, culminating in accelerated organ decline and a propensity to develop fatty liver disease at relatively early stages of life. Our findings begin to shed light on the mechanisms underlying HF-induced developmental programming of fatty liver disease and premature organ decline.

Here, we show that clock gene expression is deregulated in offspring fed a HF diet. Since SIRT1 functionally interacts with the core clock genes, it is plausible to suggest that our observed changes in NAD^+^ and sirtuin abundance play a role in deregulated clock gene expression. We show that hepatic *Bmal1* and *Cry2* gene expression are profoundly altered in offspring of HF fed mothers. It is important to highlight that the simultaneous reduction of *Cry2* and increase in *Bmal1* observed in the HF/HF offspring are directional changes that are consistent with the current model of the core-clock transcriptional feedback loops. Our findings suggest that *Bmal1* expression is influenced by nutritional challenges, and that offspring of dams fed a high fat diet, that are fed a HF diet in post-natal life have significantly up-regulated *Bmal1* expression in liver. Importantly, recent studies suggest that *Bmal1* is a novel metabolic regulator that is able to couple circadian rhythm and lipid metabolism. *Bmal1* knockout mice show both impaired insulin signaling and lipid homeostasis [45]. Recent studies have shown that mice with either a global or hepatic *Bmal1* deficiency have reduced lipogenic gene expression following re-feeding [46]. Conversely, *Bmal1* over expression in the liver is sufficient to elevate the expression of genes involved in de novo lipogenesis, such as *Srebp1c* and *Fasn* (Zhang et al. [29]). Again, this is consistent with our observations showing the concurrent increase in CCGs (*Rev-Erb*, *RORα* and *Srebp1c*) with key roles in lipid lipogenesis. Specifically, *Rev-Erbα* is known to regulate the *Srebp1c* promoter and mRNA expression [47]. Here we show that in the C/HF and HF/HF offspring we see a subtly different pattern of hepatic “circadian” *Srebp1c* gene expression, but overall the expression of *Srepb1c* was elevated compared to the control offspring, as expected in steatotic livers. Nonetheless, *Srebp1c* levels were the highest the HF/HF group. Interestingly, while there was a trend towards increased *Rev-Erbα* expression in the C/HF group, only the HF/HF group show significantly elevated expression. Thus, we hypothesize that the elevated *Rev-Erbα* may be associated with increased *Srepb1c* expression observed in the HF/HF offspring. However, we cannot rule out other possible pathways that may be contributing to the increased expression of both of these genes, following differential dietary exposures between the offspring groups. Taken together our results suggest that a deregulated core clock gene-lipogenic transcription factor axis culminates in elevated hepatic fat accumulation and disease progression (Fig. 4).

The impact of HF diets on clock gene expression and lipid homeostasis in the liver has been the subject of intense research of late. Nonetheless, controversy remains as to whether HF diets can significantly affect hepatic clock gene expression. Relatively short term HF feeding in mice produced a metabolic syndrome phenotype, but did not alter hepatic clock gene expression [48]. In contrast, recent studies have shown that HF feeding leads to changes in circadian locomotor activity, food intake, and hepatic clock gene expression [49], [50]. In addition, long term HF feeding can have a major impact on clock gene expression in the liver [51]. It is therefore possible that the length or timing of HF exposure may have variable effects on the liver. Importantly, a recent study showed that developmental HF exposure disrupts circadian rhythmicity and contributes to metabolic syndrome including hepatic steatosis in a rat model of maternal obesity [11]. Maternal obesity has also been shown to program offspring NALFD through disruption of circadian rhythms and clock gene expression in offspring livers in mice [27]. Our data are both consistent with, and extend these recent observations, which show that hyper-calorific diets in utero can permanently disrupt the chronobiological network [27]. Collectively these findings highlight the importance of the developmental period for liver development, and that nutritional exposures during this time can persistently alter clock genes to permanently alter liver metabolism and increase disease susceptibility in later life.

In conclusion, during the last decade fatty liver disease has become the most common chronic liver disease in young individuals [52]. Therapies tend to focus on treating the co-presenting metabolic imbalances such as obesity and increased BMI, and often have limited efficacy treating liver disease itself. Pharmacological interventions that target the underlying mechanism have considerable potential to prevent the onset of severe liver disease and additional features of the metabolic syndrome. Our findings highlight the importance of nutrition during early life as a promising area for intervention. Although further research in this area is warranted to fully understand the multifaceted nature of developmentally primed liver disease, our mechanistic insights suggest that supplementation with factors able to reverse the effects of depleted NAD^+^ reserves, and/or SIRT1 and SIRT3 abundance may also have the potential to alleviate the effects of developmental HF exposure, and rescue the increased susceptibility to develop severe fatty liver disease in later life. Our model provides a platform to further our understanding of the molecular pathogenesis of developmentally primed fatty liver disease, and to identify novel interventions.

The following is the supplementary data related to this article.

Supplemental Fig. 1 Abundance of a. SIRT1 and b. SIRT3 was determined by western blotting. 20 μg of total protein from (ZT8) 15 week old male offspring (N = 4) was separated by 12% SDS-PAGE. After electrophoresis protein was transferred to polyvinylidene difluoride (PVDF) membrane in 75 V wet transfer tank (Bio-rad, UK) for 90 min depending on protein size. Incubation with SIRT1 and SIRT3 primary antibodies (Cell Signalling Technology, The Netherlands) was carried overnight in 4 °C in the presence of 3% non-fat milk/TBST buffer. Membrane was washed in TBST buffer and incubated 2 h with HRP-conjugated secondary antibodies dissolved in 3% milk in TBS-T buffer. Signals were detected using immobilon western chemiluminescent HRP substrate (Millipore, Billerica, MA, USA). Western blots were visualized and analyzed using Versa Doc and Quantity One 1-D analysis software (Bio-Rad, USA). Supplemental Fig. 1

## Transparency document

Transparency document.

## Conflict of interest

There are no conflicts of interest regarding this manuscript.

## Figures and Tables

**Table 1:** Nighttime and daytime fold changes versus corresponding C/C levels.

	Daytime values vs C/C daytime values			Nighttime values vs C/C nighttime values		
C/HF	HF/C	HF/HF	C/HF	HF/C	HF/HF
*Sirt3*	− 3.81 ± 0.66⁎⁎	− 1.22 ± 0.32	− 2.19 ± 0.58	− 2.31 ± 0.38	1.41 ± 0.52	− 12.51 ± 2.06
*Sirt1*	− 5.71 ± 0.66⁎⁎⁎	− 1.97 ± 0.25⁎⁎	− 8.08 ± 1.30⁎⁎⁎	1.19 ± 0.27	1.23 ± 0.51	− 6.22 ± 2.03
*Clock*	− 1.16 ± 0.42	− 1.11 ± 0.21	− 1.05 ± 0.20	1.14 ± 0.22	− 1.59 ± 0.76	1.36 ± 0.12
*Bmal1*	3.30 ± 1.67	− 1.89 ± 0.75	22.20 ± 10.08⁎	1.45 ± 0.32	− 3.12 ± 0.86	− 1.25 ± 0.34
*Per2*	1.12 ± 0.15	− 1.28 ± 0.25	1.23 ± 0.32	1.82 ± 0.41	2.16 ± 0.48	2.25 ± 0.56
*Cry2*	1.29 ± 0.39	− 2.38 ± 0.32	− 3.65 ± 1.82	− 2.16 ± 0.61	3.65 ± 1.82	1.10 ± 0.24
*Rev-Erbα*	1.62 ± 0.37	1.46 ± 0.19	1.51 ± 0.19	1.74 ± 0.28	1.74 ± 0.60	3.37 ± 1.03
*Rev-Erbβ*	− 1.27 ± 0.14	− 1.73 ± 0.19	− 1.93 ± 0.21	1.65 ± 0.24	2.44 ± 0.68⁎	2.83 ± 0.38⁎⁎
*RORα*	− 1.71 ± 0.19⁎	− 1.75 ± 0.20⁎	1.02 ± 0.14	− 1.54 ± 0.55	1.92 ± 0.87	1.25 ± 0.21
*Srebp1c*	1.14 ± 0.28	1.73 ± 0.39	1.97 ± 0.31⁎	3.32 ± 0.69⁎⁎	1.83 ± 0.92	3.16 ± 0.49⁎

⁎ p < 0.05.

⁎⁎ p < 0.01.

⁎⁎⁎ p < 0.001 vs corresponding daytime or nighttime C/C levels.

**Table 2:** Nighttime fold changes versus daytime levels.

	C/C	C/HF	HF/C	HF/HF
*Sirt3*	− 1.52 ± 0.40	1.08 ± 0.35	1.13 ± 0.42	− 8.68 ± 2.51⁎⁎
*Sirt1*	− 2.77 ± 0.35⁎⁎	2.44 ± 0.58⁎	− 1.14 ± 0.29	− 2.13 ± 0.52
*Clock*	1.95 ± 0.53	1.93 ± 0.34	1.38 ± 0.32	2.15 ± 0.19⁎⁎
*Bmal1*	13.75 ± 3.78⁎⁎⁎	6.06 ± 1.34⁎⁎⁎	8.35 ± 2.89⁎⁎	− 2.02 ± 0.92
*Per2*	− 1.78 ± 0.18	− 1.13 ± 0.18	1.56 ± 0.42	1.03 ± 0.25
*Cry2*	− 1.68 ± 0.22	− 4.66 ± 1.30⁎	5.18 ± 1.91⁎⁎	2.40 ± 0.53⁎
*Rev-Erbα*	− 11.54 ± 2.29⁎⁎⁎	− 10.80 ± 2.46⁎⁎⁎	− 9.82 ± 1.26⁎⁎⁎	− 5.18 ± 0.64⁎⁎
*Rev-Erbβ*	− 6.73 ± 0.73⁎⁎⁎⁎	− 3.22 ± 0.84⁎	− 1.59 ± 0.44	− 1.23 ± 0.15
*RORα*	− 1.20 ± 0.14	− 1.08 ± 0.17	2.79 ± 0.80⁎⁎	1.01 ± 0.17
*Srebp1c*	− 2.48 ± 0.56	1.17 ± 0.24	1.28 ± 0.64	− 1.55 ± 0.24

⁎ p < 0.05.

⁎⁎ p < 0.01.

⁎⁎⁎ p < 0.001.

⁎⁎⁎⁎ p < 0.0001 vs corresponding daytime or nighttime C/C levels.

## References

[1] Adams L.A., Angulo P. (2005). Recent concepts in non-alcoholic fatty liver disease. Diabet. Med..

[2] Hassan K., Bhalla V., Ezz El R.M., Kader H.H. (2014). Nonalcoholic fatty liver disease: a comprehensive review of a growing epidemic. World J. Gastroenterol..

[3] Ozcan U., Cao Q., Yilmaz E., Lee A.H., Iwakoshi N.N., Ozdelen E., Tuncman G., Gorgun C., Glimcher L.H., Hotamisligil G.S. (2004). Endoplasmic reticulum stress links obesity, insulin action, and type 2 diabetes. Science.

[4] Shneider B.L., Gonzalez-Peralta R., Roberts E.A. (2006). Controversies in the management of pediatric liver disease: hepatitis B, C and NAFLD: summary of a single topic conference. Hepatology.

[5] Loomba R., Sirlin C.B., Schwimmer J.B., Lavine J.E. (2009). Advances in pediatric nonalcoholic fatty liver disease. Hepatology.

[6] Della C.C., Alisi A., Saccari A., De V.R., Vania A., Nobili V. (2012). Nonalcoholic fatty liver in children and adolescents: an overview. J. Adolesc. Health.

[7] Gluckman P.D., Hanson M.A., Beedle A.S., Spencer H.G. (2008). Predictive adaptive responses in perspective. Trends Endocrinol. Metab..

[8] Bateson P., Gluckman P., Hanson M. (2014). The biology of developmental plasticity and the predictive adaptive response hypothesis. J. Physiol..

[9] McCurdy C.E., Bishop J.M., Williams S.M., Grayson B.E., Smith M.S., Friedman J.E., Grove K.L. (2009). Maternal high-fat diet triggers lipotoxicity in the fetal livers of nonhuman primates. J. Clin. Invest..

[10] Bruce K.D., Cagampang F.R., Argenton M., Zhang J., Ethirajan P.L., Burdge G.C., Bateman A.C., Clough G.F., Poston L., Hanson M.A., McConnell J.M., Byrne C.D. (2009). Maternal high-fat feeding primes steatohepatitis in adult mice offspring, involving mitochondrial dysfunction and altered lipogenesis gene expression. Hepatology.

[11] Borengasser S.J., Kang P., Faske J., Gomez-Acevedo H., Blackburn M.L., Badger T.M., Shankar K. (2014). High fat diet and in utero exposure to maternal obesity disrupts circadian rhythm and leads to metabolic programming of liver in rat offspring. PLoS ONE.

[12] Ashino N.G., Saito K.N., Souza F.D., Nakutz F.S., Roman E.A., Velloso L.A., Torsoni A.S., Torsoni M.A. (2012). Maternal high-fat feeding through pregnancy and lactation predisposes mouse offspring to molecular insulin resistance and fatty liver. J. Nutr. Biochem..

[13] Gregorio B.M., Souza-Mello V., Carvalho J.J., Mandarim-de-Lacerda C.A., Aguila M.B. (2010). Maternal high-fat intake predisposes nonalcoholic fatty liver disease in C57BL/6 offspring. Am. J. Obstet. Gynecol..

[14] Mazzoccoli G., Vinciguerra M., Oben J., Tarquini R., De C.S. (2014). Non-alcoholic fatty liver disease: the role of nuclear receptors and circadian rhythmicity. Liver Int..

[15] Welsh D.K., Takahashi J.S., Kay S.A. (2010). Suprachiasmatic nucleus: cell autonomy and network properties. Annu. Rev. Physiol..

[16] Bass J., Takahashi J.S. (2010). Circadian integration of metabolism and energetics. Science.

[17] Lowrey P.L., Takahashi J.S. (2011). Genetics of circadian rhythms in mammalian model organisms. Adv. Genet..

[18] Albrecht U. (2012). Timing to perfection: the biology of central and peripheral circadian clocks. Neuron.

[19] Turek F.W., Joshu C., Kohsaka A., Lin E., Ivanova G., McDearmon E., Laposky A., Losee-Olson S., Easton A., Jensen D.R., Eckel R.H., Takahashi J.S., Bass J. (2005). Obesity and metabolic syndrome in circadian clock mutant mice. Science.

[20] Shimba S., Ogawa T., Hitosugi S., Ichihashi Y., Nakadaira Y., Kobayashi M., Tezuka M., Kosuge Y., Ishige K., Ito Y., Komiyama K., Okamatsu-Ogura Y., Kimura K., Saito M. (2011). Deficient of a clock gene, brain and muscle Arnt-like protein-1 (BMAL1), induces dyslipidemia and ectopic fat formation. PLoS ONE.

[21] Rey G., Cesbron F., Rougemont J., Reinke H., Brunner M., Naef F. (2011). Genome-wide and phase-specific DNA-binding rhythms of BMAL1 control circadian output functions in mouse liver. PLoS Biol..

[22] Guillaumond F., Dardente H., Giguere V., Cermakian N. (2005). Differential control of Bmal1 circadian transcription by REV-ERB and ROR nuclear receptors. J. Biol. Rhythm..

[23] Cho H., Zhao X., Hatori M., Yu R.T., Barish G.D., Lam M.T., Chong L.W., DiTacchio L., Atkins A.R., Glass C.K., Liddle C., Auwerx J., Downes M., Panda S., Evans R.M. (2012). Regulation of circadian behaviour and metabolism by REV-ERB-alpha and REV-ERB-beta. Nature.

[24] Solt L.A., Wang Y., Banerjee S., Hughes T., Kojetin D.J., Lundasen T., Shin Y., Liu J., Cameron M.D., Noel R., Yoo S.H., Takahashi J.S., Butler A.A., Kamenecka T.M., Burris T.P. (2012). Regulation of circadian behaviour and metabolism by synthetic REV-ERB agonists. Nature.

[25] Dolatshad H., Cary A.J., Davis F.C. (2010). Differential expression of the circadian clock in maternal and embryonic tissues of mice. PLoS ONE.

[26] Suter M., Bocock P., Showalter L., Hu M., Shope C., McKnight R., Grove K., Lane R., Aagaard-Tillery K. (2011). Epigenomics: maternal high-fat diet exposure in utero disrupts peripheral circadian gene expression in nonhuman primates. FASEB J..

[27] Mouralidarane A., Soeda J., Sugden D., Bocianowska A., Carter R., Ray S., Saraswati R., Cordero P., Novelli M., Fusai G., Vinciguerra M., Poston L., Taylor P.D., Oben J.A. (2015). Maternal obesity programs offspring non-alcoholic fatty liver disease through disruption of 24-hour rhythms in mice. Int. J. Obes..

[28] Bellet M.M., Orozco-Solis R., Sahar S., Eckel-Mahan K., Sassone-Corsi P. (2011). The time of metabolism: NAD^+^, SIRT1, and the circadian clock. Cold Spring Harb. Symp. Quant. Biol..

[29] Zhou B., Zhang Y., Zhang F., Xia Y., Liu J., Huang R., Wang Y., Hu Y., Wu J., Dai C., Wang H., Tu Y., Peng X., Wang Y., Zhai Q. (2014). CLOCK/BMAL1 regulates circadian change of mouse hepatic insulin sensitivity by SIRT1. Hepatology.

[30] Chalkiadaki A., Guarente L. (2012). Sirtuins mediate mammalian metabolic responses to nutrient availability. Nat. Rev. Endocrinol..

[31] Nakagawa T., Guarente L. (2014). SnapShot: sirtuins, NAD, and aging. Cell Metab..

[32] Imai S., Guarente L. (2014). NAD^+^ and sirtuins in aging and disease. Trends Cell Biol..

[33] Borengasser S.J., Lau F., Kang P., Blackburn M.L., Ronis M.J., Badger T.M., Shankar K. (2011). Maternal obesity during gestation impairs fatty acid oxidation and mitochondrial SIRT3 expression in rat offspring at weaning. PLoS ONE.

[34] He J., Hu B., Shi X., Weidert E.R., Lu P., Xu M., Huang M., Kelley E.E., Xie W. (2013). Activation of the aryl hydrocarbon receptor sensitizes mice to nonalcoholic steatohepatitis by deactivating mitochondrial sirtuin deacetylase Sirt3. Mol. Cell. Biol..

[35] Kleiner D.E., Brunt E.M., Van N.M., Behling C., Contos M.J., Cummings O.W., Ferrell L.D., Liu Y.C., Torbenson M.S., Unalp-Arida A., Yeh M., McCullough A.J., Sanyal A.J. (2005). Design and validation of a histological scoring system for nonalcoholic fatty liver disease. Hepatology.

[36] Bruce K.D., Sihota K.K., Byrne C.D., Cagampang F.R. (2012). The housekeeping gene YWHAZ remains stable in a model of developmentally primed non-alcoholic fatty liver disease. Liver Int..

[37] Vajro P., Lenta S., Socha P., Dhawan A., McKiernan P., Baumann U., Durmaz O., Lacaille F., McLin V., Nobili V. (2012). Diagnosis of nonalcoholic fatty liver disease in children and adolescents: position paper of the ESPGHAN Hepatology Committee. J. Pediatr. Gastroenterol. Nutr..

[38] Ahn B.H., Kim H.S., Song S., Lee I.H., Liu J., Vassilopoulos A., Deng C.X., Finkel T. (2008). A role for the mitochondrial deacetylase Sirt3 in regulating energy homeostasis. Proc. Natl. Acad. Sci. U. S. A..

[39] Kendrick A.A., Choudhury M., Rahman S.M., McCurdy C.E., Friederich M., Van Hove J.L., Watson P.A., Birdsey N., Bao J., Gius D., Sack M.N., Jing E., Kahn C.R., Friedman J.E., Jonscher K.R. (2011). Fatty liver is associated with reduced SIRT3 activity and mitochondrial protein hyperacetylation. Biochem. J..

[40] Bao J., Scott I., Lu Z., Pang L., Dimond C.C., Gius D., Sack M.N. (2010). SIRT3 is regulated by nutrient excess and modulates hepatic susceptibility to lipotoxicity. Free Radic. Biol. Med..

[41] Canto C., Houtkooper R.H., Pirinen E., Youn D.Y., Oosterveer M.H., Cen Y., Fernandez-Marcos P.J., Yamamoto H., Andreux P.A., Cettour-Rose P., Gademann K., Rinsch C., Schoonjans K., Sauve A.A., Auwerx J. (2012). The NAD(+) precursor nicotinamide riboside enhances oxidative metabolism and protects against high-fat diet-induced obesity. Cell Metab..

[42] Lelliott C., Vidal-Puig A.J. (2004). Lipotoxicity, an imbalance between lipogenesis de novo and fatty acid oxidation. Int. J. Obes. Relat. Metab. Disord..

[43] Wang Z., Xu M., Peng J., Jiang L., Hu Z., Wang H., Zhou S., Zhou R., Hultstrom M., Lai E.Y. (2013). Prevalence and associated metabolic factors of fatty liver disease in the elderly. Exp. Gerontol..

[44] Floreani A. (2007). Liver diseases in the elderly: an update. Dig. Dis..

[45] Rudic R.D., McNamara P., Curtis A.M., Boston R.C., Panda S., Hogenesch J.B., Fitzgerald G.A. (2004). BMAL1 and CLOCK, two essential components of the circadian clock, are involved in glucose homeostasis. PLoS Biol..

[46] Zhang D., Tong X., Arthurs B., Guha A., Rui L., Kamath A., Inoki K., Yin L. (2014). Liver clock protein BMAL1 promotes de novo lipogenesis through insulin-mTORC2-AKT signaling. J. Biol. Chem..

[47] Ramakrishnan S.N., Lau P., Crowther L.M., Cleasby M.E., Millard S., Leong G.M., Cooney G.J., Muscat G.E. (2009). Rev-erb beta regulates the Srebp-1c promoter and mRNA expression in skeletal muscle cells. Biochem. Biophys. Res. Commun..

[48] Yanagihara H., Ando H., Hayashi Y., Obi Y., Fujimura A. (2006). High-fat feeding exerts minimal effects on rhythmic mRNA expression of clock genes in mouse peripheral tissues. Chronobiol. Int..

[49] Kohsaka A., Laposky A.D., Ramsey K.M., Estrada C., Joshu C., Kobayashi Y., Turek F.W., Bass J. (2007). High-fat diet disrupts behavioral and molecular circadian rhythms in mice. Cell Metab..

[50] Challet E. (2013). Circadian clocks, food intake, and metabolism. Prog. Mol. Biol. Transl. Sci..

[51] Hsieh M.C., Yang S.C., Tseng H.L., Hwang L.L., Chen C.T., Shieh K.R. (2010). Abnormal expressions of circadian-clock and circadian clock-controlled genes in the livers and kidneys of long-term, high-fat-diet-treated mice. Int. J. Obes..

[52] Alisi A., Nobili V. (2012). Non-alcoholic fatty liver disease in children now: lifestyle changes and pharmacologic treatments. Nutrition.

[53] Catta-Preta M., Mendonca L.S., Fraulob-Aquino J., Aguila M.B., Mandarim-de-Lacerda C.A. (2011). A critical analysis of three quantitative methods of assessment of hepatic steatosis in liver biopsies. Virchows Arch..

