# Supplementary figures and images for: Altered cellular redox status, sirtuin abundance and clock gene expression in a mouse model of developmentally primed NASH
Source: Biochim Biophys Acta. 2016 Jul;1861(7):584–93. doi: 10.1016/j.bbalip.2016.03.026 (PMC4874946; doi:10.1016/j.bbalip.2016.03.026)

## Slide 1
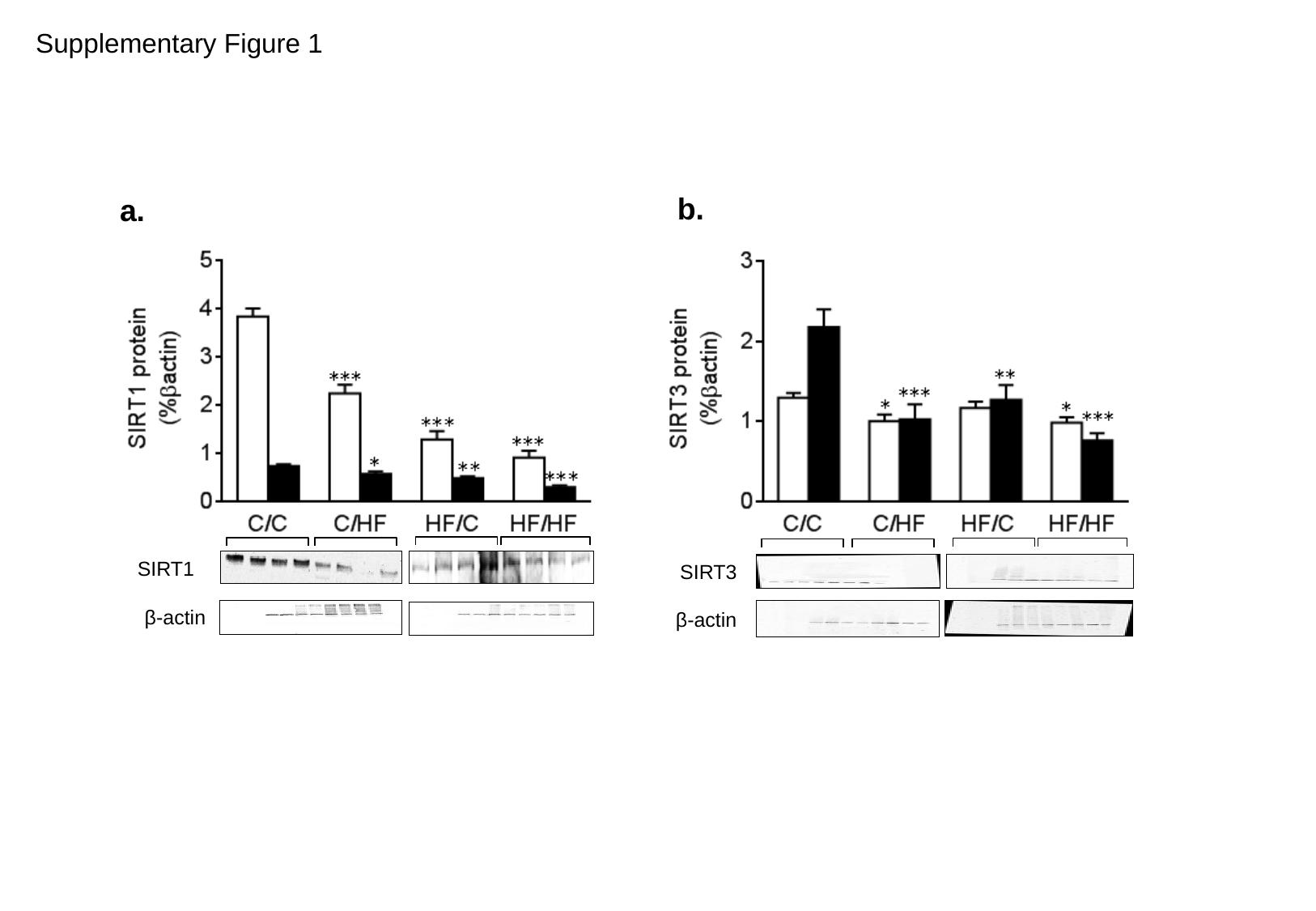

Supplementary Figure 1
b.
a.
SIRT1
SIRT3
β-actin
β-actin

Supplement: Supplemental Fig. 1 — Abundance of a. SIRT1 and b. SIRT3 was determined by western blotting. 20 μg of total protein from (ZT8) 15 week old male offspring (N = 4) was separated by 12% SDS-PAGE. After electrophoresis protein was transferred to polyvinylidene difluoride (PVDF) membrane in 75 V wet transfer tank (Bio-rad, UK) for 90 min depending on protein size. Incubation with SIRT1 and SIRT3 primary antibodies (Cell Signalling Technology, The Netherlands) was carried overnight in 4 °C in the presence of 3% non-fat milk/TBST buffer. Membrane was washed in TBST buffer and incubated 2 h with HRP-conjugated secondary antibodies dissolved in 3% milk in TBS-T buffer. Signals were detected using immobilon western chemiluminescent HRP substrate (Millipore, Billerica, MA, USA). Western blots were visualized and analyzed using Versa Doc and Quantity One 1-D analysis software (Bio-Rad, USA). [file mmc1.pptx]
